# Evaluation and Acceptability of a Simplified Test of Visual Function at Birth in a Limited-Resource Setting

**DOI:** 10.1371/journal.pone.0157087

**Published:** 2016-06-14

**Authors:** Verena I. Carrara, Mue Chae Darakomon, Nant War War Thin, Naw Ta Kaw Paw, Naw Wah, Hser Gay Wah, Naw Helen, Suporn Keereecharoen, Naw Ta Mlar Paw, Podjanee Jittamala, François H. Nosten, Daniela Ricci, Rose McGready

**Affiliations:** 1 Shoklo Malaria Research Unit, Mahidol-Oxford Tropical Medicine Research Unit, Faculty of Tropical Medicine, Mahidol University, Mae Sot, Thailand; 2 Department of Tropical Hygiene, Faculty of Tropical Medicine, Mahidol University, Bangkok, Thailand; 3 Shoklo Malaria Research Unit, Mahidol-Oxford Tropical Medicine Research Unit, Faculty of Tropical Medicine, Mahidol University, Mae Sot, Thailand; Centre for Tropical Medicine and Global Health, Nuffield Department of Medicine, University of Oxford, Oxford, United Kingdom; 4 Paediatric Neurology Unit, Catholic University, Rome, Italy; National Eye Institute, UNITED STATES

## Abstract

Neurological examination, including visual fixation and tracking of a target, is routinely performed in the Shoklo Malaria Research Unit postnatal care units on the Thailand-Myanmar border. We aimed to evaluate a simple visual newborn test developed in Italy and performed by non-specialized personnel working in neonatal care units. An intensive training of local health staff in Thailand was conducted prior to performing assessments at 24, 48 and 72 hours of life in healthy, low-risk term singletons. The 48 and 72 hours results were then compared to values obtained to those from Italy. Parents and staff administering the test reported on acceptability. One hundred and seventy nine newborns, between June 2011 and October 2012, participated in the study. The test was rapidly completed if the infant remained in an optimal behavioral stage (7 ± 2 minutes) but the test duration increased significantly (12 ± 4 minutes, p < 0.001) if its behavior changed. Infants were able to fix a target and to discriminate a colored face at 24 hours of life. Horizontal tracking of a target was achieved by 96% (152/159) of the infants at 48 hours. Circular tracking, stripe discrimination and attention to distance significantly improved between each 24-hour test period. The test was easily performed by non-specialized local staff and well accepted by the parents. Healthy term singletons in this limited-resource setting have a visual response similar to that obtained to gestational age matched newborns in Italy. It is possible to use these results as a reference set of values for the visual assessment in Karen and Burmese infants in the first 72 hours of life. The utility of the 24 hours test should be pursued.

## Introduction

When visual assessment of neonates is tested in limited-resource settings it is constrained to the observation of ocular movements and the ability to fix and follow a target during the routine newborn examination. Further detailed visual assessment requires time, specific training and is often performed at a later age when the newborn has left the post-natal ward and, typically, such tests are not available to most low-income countries. Recent work conducted in two Italian neonatal units has established and validated a battery of tests to evaluate a larger range of visual function of the newborn [1, 2]. This evaluation is short and simple enough to be performed by non-specialized health staff who can incorporate it into the routine newborn examination. The test tools are simple, light, portable and washable targets, which do not require bulky, expensive equipment or specific settings making them ideal for limited-resource settings. Neonatal visual function was explained in the seventies by Bronson who suggested a model for human visual development in which newborn vision is mainly controlled at a subcortical level [3]. The relevance of such subcortical control was later confirmed by imaging studies showing normal ability to fix and follow an object in infants with extensive occipital lesions above the subcortical level [4]. The battery of tests proposed by Ricci *et al*. was the first structured examination assessing different aspects of visual function within the early neonatal period; its use to assess the visual function of term infants and of infants born at less than 31 weeks of gestation showed three different trends in visual development; items such as fixation to and horizontal tracking a target are mature as early as 35 weeks postmenstrual age while ocular motility or tracking vertically and in circle are influenced by the extra-uterine experience. Color and stripe discrimination as well as attention to distance require a certain brain maturity and are more mature at term gestation [5].

The Shoklo Malaria Research Unit (SMRU) has conducted routine newborn neurological examination since 1995 using a simplified validated version of the neurological assessment developed by L. Dubowitz [6]. As part of this assessment a red woolen ball and a black and white target were used to assess the newborn’s ability to focus on, fixate and track an object, horizontally, vertically and in a circle. Using these tools, the visual performance at birth of 38 infants born in the Karen refugee camp (March 1996) was poor compared to British and Thai newborns but vision normalized within the first 6 months of life [7]. This delay was postulated to be associated with deficiencies of micronutrients in pregnancy, in particular thiamine due to its relation to neuronal myelination [8], or combined with high levels of exposure to dichlorodiphenyl trichloroetane (DDT) which have been reported to induce thiamine deficiency in animals [9]. Since this first study was conducted thiamine hydrochloride (vitamin B1) supplements have been routinely supplied to all pregnant and lactating women and deficiencies have become rare [10]. In addition the yearly programme of indoor-residual spraying for malaria control ended in 2000, reducing exposure to pesticides.

The main objective of this study was to evaluate the visual performance of healthy, normal, low-risk, term singletons born to mothers attending antenatal care in the SMRU clinics serving a refugee and a migrant population along the Thailand-Myanmar border with the battery of test items described by Ricci *et al*. [1]. The secondary aims were to compare the range of visual responses at 48 and 72 hours of life with previously published results [2] and, in a subset of newborns, to perform a visual assessment at 24 hours of life as it is likely to be more applicable in low-income countries with high rates of early neonatal discharge.

## Methods

### Study site and population

The study was carried out in two birth units run by the SMRU along the Thailand-Myanmar border. One was located in Maela refugee camp, 60 km north of the Thai township of Mae Sot, the other in Mawker Thai village, 60 km south of Mae Sot; Maela refugee camp hosts 50,000 displaced people from neighboring Myanmar, whilst Mawker Thai clinic serves migrant workers also from Myanmar. The population in both areas is mostly from the Karen ethnic minority, the living conditions are fairly similar with housing made of wood, bamboo and the roof made of leaves [11]; half of the pregnant women attending the clinics are literate [12].

Women are encouraged to follow antenatal care (ANC) as soon as they are aware of their pregnancy and to deliver with skilled birth attendants at the SMRU facility [13]. While following ANC they are offered early detection and treatment of malaria as there are no other reliable measures to reduce maternal mortality due to malaria in this area of highly *P*. *falciparum* multidrug resistant strains [13]. Other care includes anemia prophylaxis, thiamine (vitamin B1) supplementation [8], tetanus vaccination [14], hepatitis B and syphilis screening and prevention of maternal-child transmission of HIV [15]. Gestational age of the pregnancy is determined by ultrasound performed by local health-workers [16] or Dubowitz examination [17]. The clinic is open on a 24-hour basis, for birthing services (no anesthesia) and a special care baby unit (SCBU). The SCBU provides essential neonatal care but is a facility without capacity for ventilation [18].

### Study design

The study was conducted between June 2011 and October 2012 and was approved by the Oxford Tropical Research Ethics Committee (OXTREC 15–11) and by the Ethics Committee of the Faculty of Tropical Medicine, Mahidol University (MUTM 2011-011-01). Parents of eligible newborns were explained the purpose of the study and if written informed consent was forth coming their newborn was included.

As the study aimed at evaluating the visual function of a low-risk newborn population, only singleton normal term babies born to women with regular antenatal care, an uneventful pregnancy and a normal vaginal delivery were considered eligible. Using those eligibility criteria, we aimed at obtaining a low-risk birth cohort as similar as possible to the Italian cohort [2]. Clear differences in maternal demographics and newborn birthweights were expected as high- and low-income cohorts were being compared. Newborns who presented with signs of fetal distress, had birth resuscitation or an Apgar score < 7 at 5 min, needed oxygen therapy, developed jaundice requiring phototherapy, had an eye infection or abnormal clinical findings requiring an hospitalization to the special care baby unit (SCBU) were not enrolled in the study. Parents of newborns who could participate in the study were asked about their socio-demographic characteristics. Acceptability of the newborn visual assessment was evaluated by asking a parent to score on a 0–10 scale the following: length of the test, its usefulness, whether they felt it was safe for the child and if they would bring the infant back if asked again, or would encourage other parents to come for such an assessment. Informal individual interviews with open questions were conducted among the local health staff performing the test to evaluate their perception of the test.

### Training and inter-observer reliability

Prior to starting the study the senior examiner (DR), from the Pediatric Neurology Unit of the Catholic University of Roma, Italy, conducted a 2-week training in Maela camp. One expatriate doctor (VIC) and 4 local health staff participated in the training. Two trainees had previous experience with some aspects of newborn visual assessment and neurological examination. Training included instructions on how to use each of the battery of items and practice sessions to get familiarized with the tools and to perform tasks uniformly. The inter-observer reliability was then assessed by asking each trainee to comment and score on newborn tests performed by each other until the concordance between observers reached ≥ 95%.

### Test procedure

**Fig 1:**
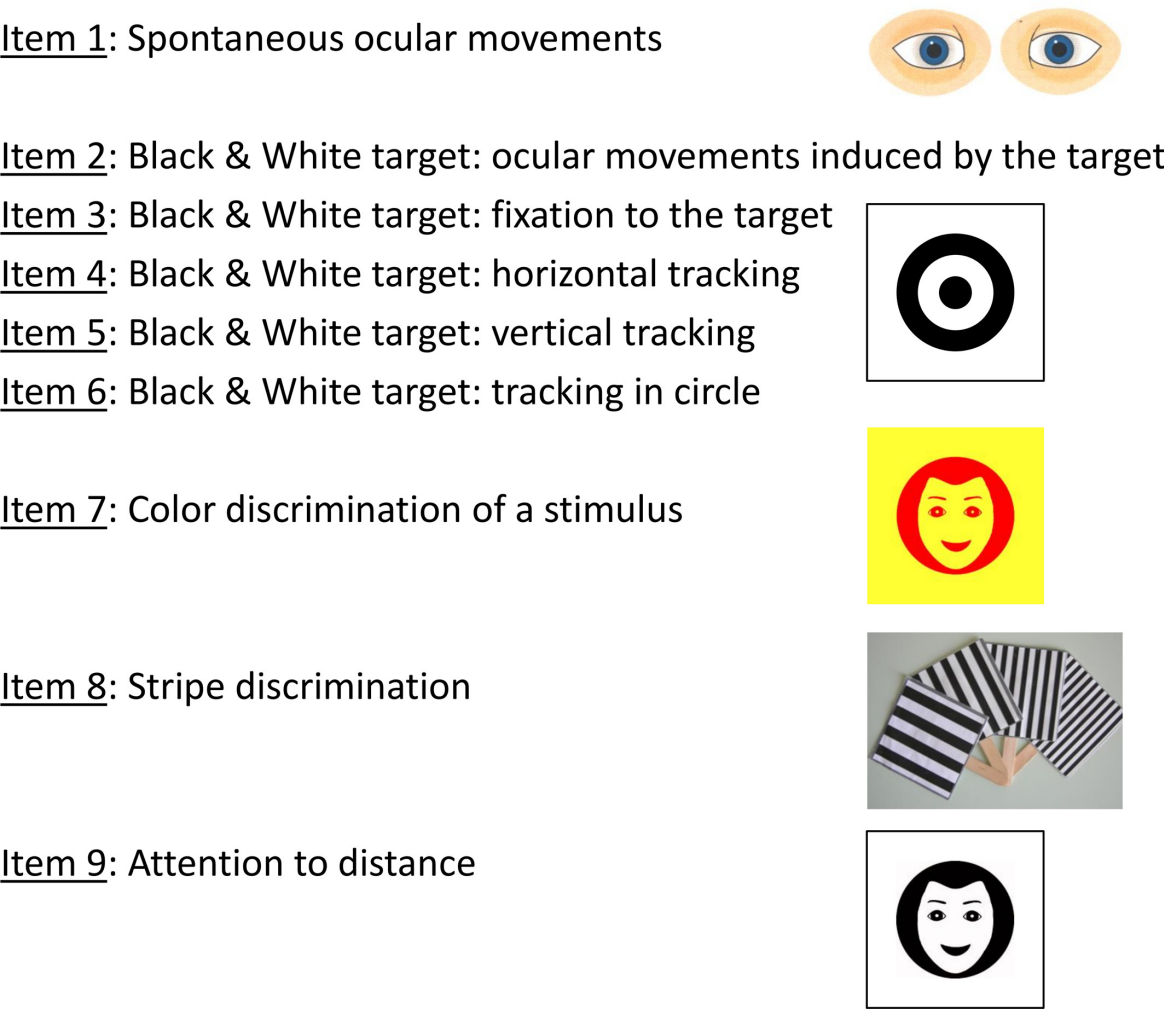
The nine items included in the visual assessment battery. Four different stimuli were used: a black and white target; a yellow and red colored face; a black and white target and 8 cards with stripes of different spatial frequency. Ocular movements were observed when the newborn was resting and while tracking with the black and white target. (Credit to D. Ricci)

Prior to testing, newborns were weighed, and their clinical condition evaluated. Newborns were assessed at 24 ± 8 hours (if parental agreement was obtained early enough), at 48 ± 8 hours and at 72 ± 8 hours using the 9 item visual assessment battery (including ocular motility, fixation to and tracking a target, color and stripe discrimination, and attention at a distance) (**Fig 1**) [2]. As there were no comparative data available for the visual assessment of newborns at 24 hours of age it was agreed to evaluate whether it was possible to obtain a complete test. The concern was that the behavior of the child in this early neonatal period could result in an inability to complete the test items compared to the results that could be obtained at 48 and 72 hours. In the context of a population used to early discharge from the birthing unit the first 24 hours test has the potential to be more widely applicable.

Visual assessment was performed in an appropriately lit room nearby the post-natal care station, in a quiet environment, and in the presence of a parent. The newborn was held propped, in a semi-supine position; talking and other auditory stimuli were maintained to a minimum while presenting the visual stimuli to the newborn. One tester and one observer were present each time; tests results were reported on a pro-forma adapted for this setting: wording was simplified and pictures were added to improve the comprehension of health staff where the use of the English language was their 3^rd^ or 4^th^ language. Incomplete visual assessment and changes of behavioral state during the test were recorded. The duration of the test was timed from starting the first test item until completion of all 9 items; it did not include the time to awaken the newborn prior to starting the test. In the infrequent case where the tester and observer wanted to assign a different result on the pro-forma that could not be resolved by discussion, the item could be rechecked.

### Statistical analysis

All information recorded in the clinical record form, the visual pro-forma and the acceptability questionnaire was double data entered in an access database (Microsoft Office Access 2007) and discrepancies between the datasets were checked against the hard copy; additional maternal data was extracted from the antenatal care computerized database. All data were analyzed in SPSS for Windows version 14.0 (SPSS, Inc., Chicago, Illinois, USA). Continuous normally distributed data were described by their mean and their standard deviation (SD), non-normally distributed data by their median and range. Proportions were used to describe categorical data. Demographic and socio-economic characteristics of parents and newborns (Maela refugee camp families *vs*. migrant families from Mawker Thai clinic) were compared using the Chi-square test with Yates’ continuity correction or the Fisher's exact test for the categorical variables and the independent student’s t-test or the Mann-Whitney U test for continuous variables. Paired t-test or Wilcoxon signed-rank test were used for comparing the variables of the visual assessments at 24, 48 or 72 hours. A p-value of < 0.05 was considered significant. Maternal and newborn individual patient data from the Italian cohort were not available for analysis; only variables presented in the published manuscript could therefore be compared (mean birthweight, mean gestational age at birth and visual assessment items percentages).

## Results

A total of 203 newborns were enrolled in the study, 34 in Mawker Thai and 169 in Maela. Twenty-four newborns were excluded from further analysis: two were non-eligible (one had basic newborn resuscitation, one had a congenital abnormality), fifteen were unexpectedly hospitalized to the SCBU after the consent for the study had been given, two had acute conjunctivitis within the first 72 hours of life, three did not come to either appointment, and two failed to complete any visual assessments. The analysis was done for the remaining 179 newborns who completed at least one visual assessment. Demographic and socio-economic characteristics of their parents are presented in **S1 Table**. As expected the duration of residence at the current address along the Thailand-Myanmar border was shorter for migrants than for families living in the camp (a median of 6.0 *vs*. 10.0 years for mothers, p = 0.059 and of 6.5 *vs*. 11.5 years for fathers, p = 0.006) and maternal literacy was higher in the refugee population (109/149, 73.2% vs. 16/30, 53.3%, p = 0.048). None of the pregnant women had malaria; pregnancy characteristics were similar in refugee and migrant populations. There was a similar proportion of women underweight (BMI < 18.5 kg/m^2^) and of women with BMI ≥ 23.0 kg/m^2^, the cut-offs for being at health risk for obesity-related complications in Asian populations [19]. Characteristics of the 179 newborns who completed at least one visual assessment are presented in **Table 1**. Female newborns were significantly shorter (49.2cm *vs*. 50.1 cm, p < 0.001) and lighter (2978 g *vs*. 3108 g, p = 0.017) than males at birth; the difference in weight remained significant at 48 and 72 hours of life, however the percentage of weight change was similar for both genders.

**Table 1:** Characteristics of 179 newborns (87 males and 92 females).

Characteristics	Male	Female	P value
EGA (weeks)	39.3 ± 0.85 [37.4–41.1]	39.2 ± 0.86 [37.2–41.4]	0.528
Apgar score at 1 minute	9 [7–10]	9 [8–9]	0.404
Apgar score at 5 minutes	10 [9–10]	10 [9–10]	0.267
Birth HC (cm)	32.2 ± 1.1 [30.0–35.0]	32.1 ± 1.2 [30.0–36.0]	0.663
Birth length (cm)	50.1 ± 1.5 [46.0–54.0]	49.2 ± 1.6 [45.5–53.5]	**<0.001**
Birth weight (g)	3108 ± 368 [2110–4080]	2978 ± 348 [2250–3920]	**0.017**
< 2,500g	5 (5.7%)	6 (6.5%)	1.000
Absent at H48	5 (5.7%)	7 (7.6%)	0.768
Absent at H72	6 (6.9%)	9 (9.8%)	0.593
H48 weight (g)	2965 ± 351 [2020–3920]	2845 ± 343 [2150–3700]	**0.027**
H72 weight (g)	2999 ± 361 [2000–3980]	2886 ± 342 [2190–3750]	**0.042**
Weight change H48—birth (%)	-4.6%	-4.5%	0.751
Weight change H72—birth (%)	-3.2%	-3.4%	0.705
Jaundice present at H48^*^	36/82 (43.9%)	50/85 (58.8%)	0.064
Jaundice present at H72^*^	58/81 (71.6%)	58/83 (69.9%)	0.865

EGA: Estimated Gestational Age; HC: head circumference; H48: 48 hours of life; H72: 72 hours of life

Numbers are mean ± SD, [range] or median [range] or number (%)

*Medical staff used Kramer zone to evaluate the jaundice intensity; Serum Bilirubin Level (SBR) measurement was done when necessary as per SMRU pediatric guidelines

### Visual assessments at 48 and 72 hours and comparison with previously published data

Twelve infants missed the 48 hours appointment and 15 were absent for the 72 hours appointment. Eight infants (8/167, 4.8%) did not complete the 48 hours test and another 8 (8/164, 4.9%) failed to complete the 72 hours test because of a change in their behavioral state; they were either irritable and could not be calmed down (n = 11) or fell asleep and could not be awakened enough to complete the assessment (n = 5). There was no significant difference in sex, age at testing, weight, or between infants who completed the test and those who did not; slight to moderate jaundice was however more often reported among those not completing the 48 (7/8, 87.5% *vs*. 79/159, 49.7%, p = 0.065) or 72 hours test (7/8, 87.5% *vs*. 109/156, 69.9%, p = 0.439).

The test was rapidly completed if the infant remained in an optimal behavioral stage (7 ± 2 minutes); its length significantly increased (12 ± 4 minutes, p < 0.001) if the infant became sleepier (n = 98) or started to cry (n = 74). All infants were able to fix a target and to discriminate a colored face (**Table 2**). Tracking horizontally and vertically a black and white target was achieved by most of the infants during the 48 hours assessment; however vertical tracking was significantly better if the behavioral state remained unchanged (64 complete tracking/80 tests, 80% *vs*. 50/78, 64% among infants with behavioral changes, p = 0.033). Stripe discrimination to a spatial frequency of ≥ 0.86 cycles per cm (4^th^ card seen) was reached by 92% and 98% of infants at 48 and 72 hours, respectively. The three infants who continued to have poor stripe discrimination score at 72 hours were all reported as being sleepy or agitated during the test. Only one infant had an attention to distance less than 30 cm during the 48 hours test, which improved the following day (43 cm).

**Table 2:** Completed visual assessment at 48 hours (H48) and 72 hours (H72).

	H48 (n = 159)	H72 (n = 156)
**Ocular motility**		
Spontaneous conjugated eye movements^*^	135 (86%)	138 (90%)
Conjugated eye movements following a target	55 (34%)	77 (50%)
**Fixation**		
Stable (> 3 sec)^*^	159 (100%)	155 (100%)
**Tracking a black & white target**		
Complete horizontal tracking	152 (96%)	153 (98%)
Complete vertical tracking^*^	114 (72%)	132 (85%)
Complete tracking in circle	91 (57%)	120 (77%)
**Tracking a colored stimulus**		
Present	159 (100%)	156 (100%)
**Stripe discrimination**		
1–3 stripes (0.32–0.64 c/cm)	12 (8%)	3 (2%)
4 stripes (0.86 c/cm)	45 (28%)	18 (12%)
5–6 stripes (1.3–1.6 c/cm)	65 (41%)	58 (37%)
7–8 stripes (2.4–3.2 c/cm)	37 (23%)	77 (49%)
**Attention to distance**		
< 30 cm	1 (1%)	0 (0%)
30–50 cm	62 (39%)	26 (17%)
51–69 cm	52 (33%)	64 (41%)
≥ 70 cm	44 (27%)	66 (42%)

Numbers are number (%)

*Spontaneous conjugated eye movements data missing for 1 newborn at H48, and 3 newborns at H72; Fixation data missing for 1 newborn at H72; Vertical tracking data missing for 1 newborn at H48

These results were compared with previously published data obtained among 110 infants born in the Gemelli hospital in Roma (**Table 3**) [2]. The estimated gestational age of both populations was similar (39.4 ± 1.1 weeks in Gemelli *vs*. 39.2 ± 0.9 in SMRU, p = 0.086), but mean birth weight was significantly higher among the Italian newborns (3450g ± 489 *vs*. 3041g ± 363, p < 0.001). Proportions of newborns able to: fix (100% at 48 hours in SMRU *vs*. 99%, p = 0.770 and 100% at 72 hours in both settings), track a black and white target (horizontal tracking at 48 hours 96% *vs*. 100%, p = 0.086 and at 72 hours 98% *vs*. 100%, p = 0.732; vertical tracking at 48 hours 72% *vs*. 73%, p = 0.967 and at 72 hours 85% *vs*. 92%, p = 0.303) or a colored target (100% at 48 hours in both settings) and their attention to the distance were similar in both cohorts (99% *vs*. 100% attention at 30+ cm at 48 hours, p = 0.808 and 100% at 72 hours in both settings). Tracking in a circle was more often considered complete at 48 hours in the SMRU setting (57% *vs*. 41%, p = 0.014), but the significance of this difference disappeared at 72 hours (77% *vs*. 66%, p = 0.173). On the other hand, poorer stripe discrimination (<0.86 c/cm) was more often reported among the 48 hours old infants born at SMRU (8% *vs*. 1%, p = 0.023), but again this difference disappeared at 72 hours of age (2% *vs*. 0%, p = 0.732). Spontaneous occasional abnormal eyes movements were less frequently observed by the SMRU team (15% *vs*. 38% at 48 hours test and 10% *vs*. 36% at 72 hours test, p < 0.001 for both) however the proportion of newborns with unconjugated eyes movements while following a moving target was similar to that of the Italian newborns (65% *vs*. 65% at 48 hours test, p = 0.897 and 50% *vs*. 56% at 72 hours test, p = 0.565).

**Table 3:** Comparison of results of the visual assessments at H48 and H72 obtained in SMRU Clinics with those previously published from Gemelli Hospital (Italy).

	H48	H48	P value	H72	H72	P value
	SMRU (n = 159)	Gemelli (n = 110)		SMRU (n = 156)	Gemelli (n = 50)	
**Ocular motility**						
Spontaneous conjugated eye movements	135/158 (85%)	68 (62%)	<0.001	138/153 (90%)	32 (64%)	<0.001
Conjugated eye movements following a target	55 (35%)	39 (35%)	0.897	77/155 (50%)	22 (44%)	0.565
**Fixation**						
Stable (> 3 sec)	159 (100%)	109 (99%)	0.770	155/155 (100%)	50 (100%)	NA
**Tracking a black & white target**						
Complete horizontal tracking	152 (96%)	110 (100%)	0.086	153 (98%)	50 (100%)	0.732
Complete vertical tracking	114/158 (72%)	80 (73%)	0.967	132 (85%)	46 (92%)	0.303
Complete tracking in circle	91 (57%)	45 (41%)	0.014	120 (77%)	33 (66%)	0.173
**Tracking a colored stimulus**						
Present	159 (100%)	110 (100%)	NA	156 (100%)	50 (100%)	NA
**Stripe discrimination**						
1–3 stripes (0.32–0.64 c/cm)	12 (8%)	1 (1%)	0.023	3 (2%)	0 (0%)	0.732
≥ 4 stripes (≥ 0.86 c/cm)	147 (92%)	109 (99%)	0.023	153 (98%)	50 (100%)	0.732
**Attention to distance**						
< 30 cm	1 (1%)	0 (0%)	0.808	0 (0%)	0 (0%)	NA
≥ 30 cm	158 (99%)	110 (100%)	0.808	50 (100%)	50 (100%)	NA

Data from Gemelli Hospital reported with the permission of D. Ricci

### Visual assessment at 24 hours and changes over time in infants with paired assessments

An additional visual assessment in the first 24 hours of life was obtained from 58 newborns, of whom four (6.9%) could not complete the test. Mean age at time of assessment was 21.8 ± 7.2 hours. The duration of the test was also 7 ± 2 minutes if the infant remained in an optimal behavioral stage and increased to 9 ± 2 minutes if there was a change in behavior (p = 0.059). All infants were able to fix a target and to discriminate a colored face at 24 hours and complete horizontal tracking was also achieved by most (53/54, 98%).

**Fig 2:**
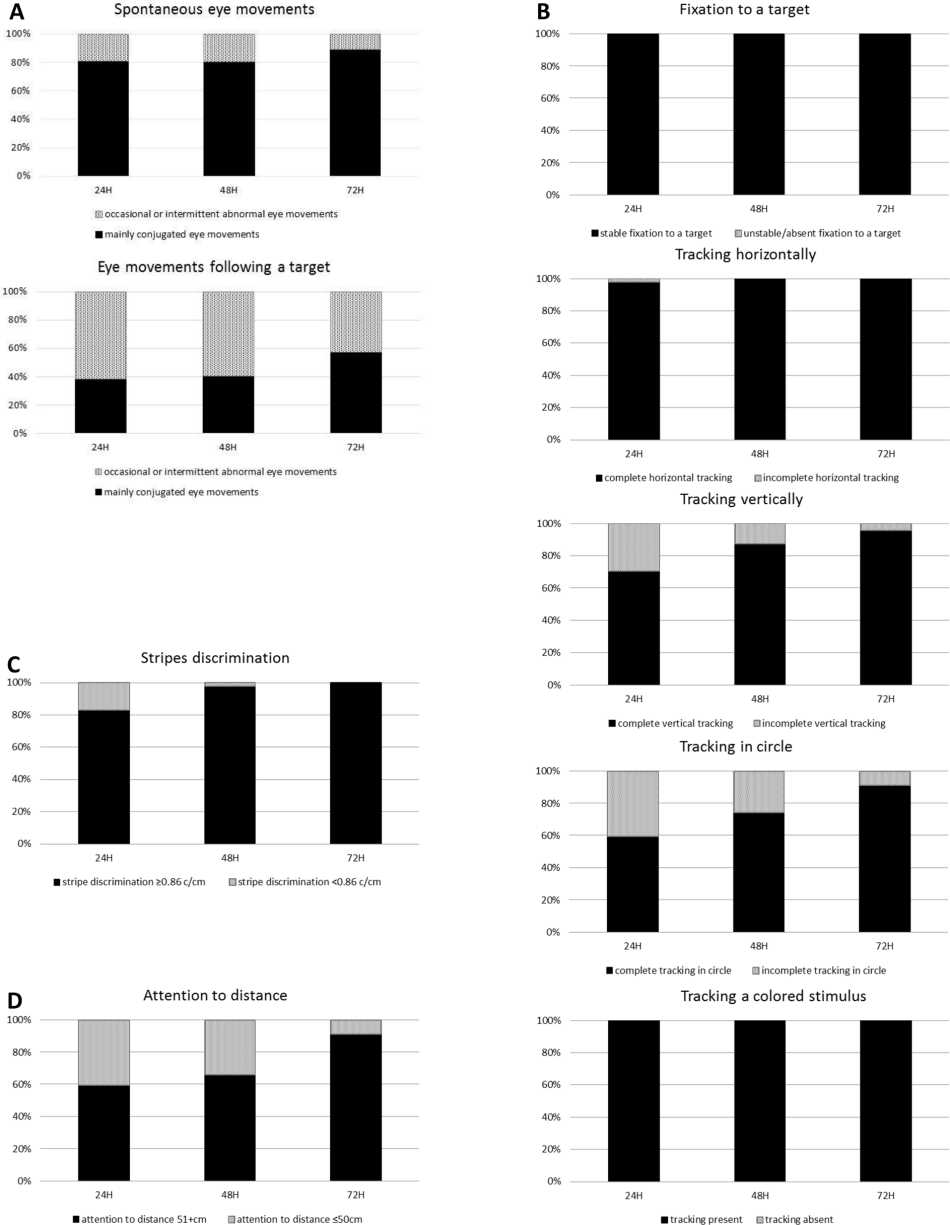
Changes in visual assessments in 47 infants with paired tests at 24, 48 and 72 hours. Changes between the first visual assessment at 24 hours (24H), the second (48H) and the last (72H) in: A- the proportion of newborns with conjugated eye movements (black bars) and occasional or intermittent abnormal eye movements (dotted bars) observed spontaneously (upper graph) and while following a target (lower graph); B- the proportion of newborns with a stable fixation to a target (upper graph), a complete tracking: horizontally (second graph), vertically (third graph), in a circle (fourth graph) and of a colored stimulus (lower graph); C- the changes in stripe discrimination and visual acuity; D- the changes in attention to distance.

Forty-seven infants completed the series of three tests and their performance at each assessment is presented in **Fig 2**. Abnormal eyes movements induced by a target were less and the overall test performance improved gradually as the infant matured despite a higher rate of behavioral changes during the later assessments. Fixation to a target, horizontal tracking and color discrimination were achieved at 24 hours of life and three items improved significantly between each of the three periods namely: tracking in a circle, stripe discrimination and attention to distance (non-parametric test for related samples, p < 0.001 for all).

Changes in performance in visual assessment between 48 and 72 hours were evaluated in another 89 infants who completed both tests and confirmed improvement in the ability to track a target vertically and in circle, stripe discrimination, and to have better attention at a distance.

### Acceptability of the visual assessment by the infant’s parents

**Fig 3:**
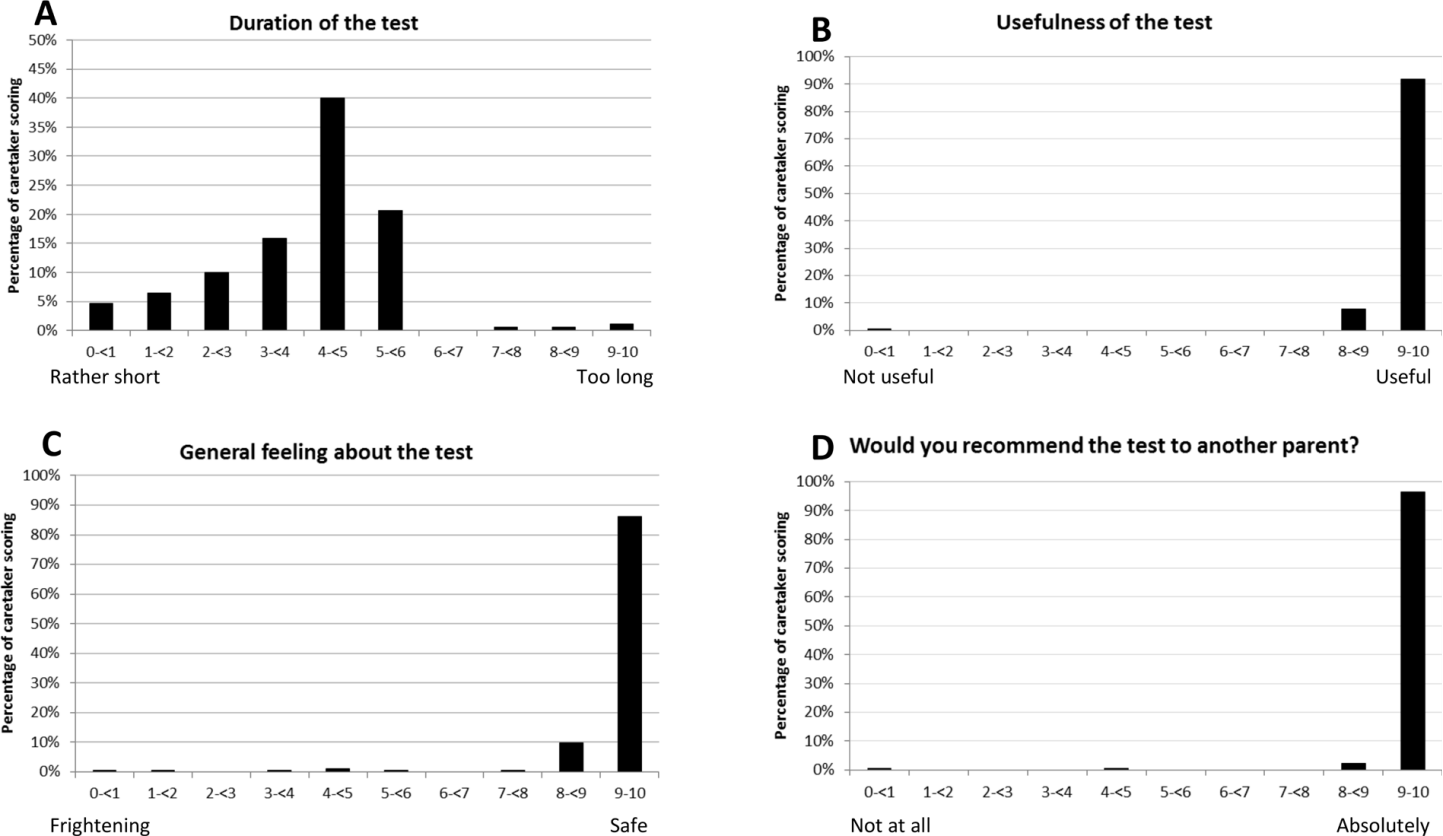
Distribution of the scores reported by the caregivers regarding the acceptability of the visual assessment. Four questions were presented on a scale from 0 to 10: duration of the test (panel A) from rather short to too long; usefulness of the test (panel B) from not useful at all to useful; general feeling about the test (panel C) from frightening to safe, and willingness to come back for another test or to recommend the test to other parents (panel D) from not at all to absolutely.

Acceptability of the test was assessed once; questionnaires were completed by 173 parents or guardians (96.6%) including 53 mothers (30%), 65 fathers (36%), and 55 other guardians, usually a close relative (31%). In 6 cases (3%), the baby’s parent was absent during the visual assessment. One parent had no opinion about the test at all. Responses of the caretakers are presented in **Fig 3**. Duration of the visual assessment was considered acceptable by 76% (130/170) of the parents; 36 (21%) thought it was rather short, while 4 (2%) thought it was too long. Three parents (2%) had no opinion on the matter. There was no correlation between the length of the assessment (in minutes) and the reported estimates by the parents. Most parents thought the test was useful (168/169, 99%) and non-frightening (165/172, 96%), while 5 (3%) felt it was only just acceptable, and two parents (2/172, 1%) considered the handling of the baby with one arm holding the baby while presenting the targets with the other hand frightening. Additionally, when asked if there were some parts of the tests that were inappropriate ten parents (10/167, 6%) suggested that the test was conducted at a too early age. Two parents reported they were not willing to come back for another visual assessment or to encourage other parents to test their newborn: for one caregiver the test was considered useless and the second lived too far to consider coming back.

### Perception of the visual assessment by the local health staff performing the test

The eight local health staff who participated in this study as well as 3 persons trained at a later stage agreed to discuss their perception of the test (**S1 Document**). The duration of the test was reported as satisfactory by most (10/11, 91%) and one tester reported it to be rather short. It was considered safe (2/11, 18%) and very safe (9/11, 92%) for the newborn. All the testers thought the test was useful not only for the newborn health but also because it gave parents a chance to observe their offspring performing the test. When asked at what time they preferred to assess the child and why, the general consensus was that it was easier to perform the test earlier in life than at 72 hours of life because the newborn was usually more alert and awake. All agreed that the most difficult and less liked part of the test was to obtain an ideal behavioral state for neonate including: to having the baby sufficiently awake to start the test, to keep the baby awake for the duration of the test, or to deal with the changes in behavior during testing.

## Discussion

Short of sending local health workers from a low-income setting on the Thailand-Myanmar border to a high-income setting in Italy, this study suggests that similar results can be obtained for the visual assessment within 72 hours of life in healthy neonates. Despite expected differences in birthweight between the settings, accurate assessment of gestational age amongst other criteria, provided the possibility to compare low-risk cohorts. Indeed the importance of postmenstrual age, rather than birth weight, to determine the maturation of the cortical aspects of visual function has been previously described [5, 20].

These are important results for this deprived population compared to a previous report documenting delayed visual maturation 20 years previously [7]. Delayed visual maturation is an early sensory deficit that can potentially lead to disruption of the normal sequence and timing of early childhood development [21] and is suspected to be caused by nutritional deficiency, most likely thiamine [7, 8], and possibly in combination with high serum dichlorodiphenyl trichloroethane (DDT) concentrations [22]. Reduction in conflict in the area has created more stable living conditions on the border. This has had positive gains for health in the community including less malaria [23] and anemia [11] and the opportunity to provide pregnant women with a steady supply of vitamins during pregnancy [12]. It is also likely that women have less exposure to DDT as its use in household spraying program was phased down in Thailand in the late 1990’s [24] with a lower possibility for thiamine deficiency induced by DDT [9]. Gains in thiamine status have been demonstrated biochemically and the pregnant and lactating women in the area have been found to be compliant with the large doses of thiamine prescribed [10, 25]. Although there were more visual items tested in the present study compared to previous studies conducted in the Thailand-Myanmar border population [7, 8], fixation and tracking of a black and white target was obtained using a very similar tool and assessed with the same technique, and it is therefore unlikely that the difference in tests explains the improvement.

The test items were easily understood and adequately performed by health staff following short intensive training; however the quality and the duration of the test, and thus the scoring, were affected by the behavioral state of the infant. Although the proportion of newborns who could not complete the visual assessment because of a change in their behavioral status was relatively low (less than 5% at either time-period) and comparable with that of the Italian cohort, the most difficult challenge reported by the testing team was to have the newborn sufficiently awake prior to testing. This is a challenge in this population where newborns are systematically swaddled to calm them and are breastfed as soon as they stir and cry [26]. Two additional factors, jaundice and weight loss, could have negatively influenced the behavior of the newborn in the 72 first hours of life; however weight loss in the first 72 hours was 3 to 4% and within the expected range for this population [27]; two-third of newborns presented with visible jaundice by the third day of life but this was mild and did not require phototherapy. The single epicanthal folds of Asian eyes might have negatively influenced observation of spontaneous ocular movements. However, this is unlikely as the reporting of occasional abnormal eye movements while following a target was similar to that of the Italian study.

Observations of visual performance at 24 hours were surprisingly good with more newborns alert and less changes in behavior compared to the testing at 48 and 72 hours. These findings mirror the responses of the local health staff preferring to do an early test as the newborn was easier to wake up or to maintain in an ideal behavioral stage. This may differ from high income settings due to the high rate of natural birth and zero use of anesthesia during childbirth in this setting. All the newborns, including those tested within 24 hours of life, had already a stable fixation and were able to track a colored stimulus. Complete horizontal tracking was achieved by most. This suggests that it might be possible to use the 24 hours data as a reference in this or other limited-resource settings with low rates of labor analgesia and where early discharge is common. Significant improvement in circular tracking, stripe discrimination and attention at distance observed between 24, 48 and 72 hours, suggests a rapid adaptation of the visual system and these need to be controlled for with this test. Further data collection to determine the utility of the 24 hours test should be encouraged.

The overall acceptability of the test reported by parents and local health staff performing it, was high. Adjustment of the one-hand holding technique in order to respond to the parents’ concerns by sitting cross-legged while holding the newborn over the tester’s lap have been incorporated into routine practice with the test.

## Conclusions

This study has two important conclusions. Firstly, the data suggests that delayed visual maturation is no longer a problem in the infants of refugee parents on the Thailand-Myanmar border and there is now a reference set of values for visual assessment in Karen and Burmese infants in the first 72 hours of life. Secondly, the test is easily performed by non-specialized health staff from the local communities, is inexpensive, and acceptable to parents. It could be added to the routine newborn examination or be included as part of research projects where vision is of interest because of in-utero exposure to infectious agents or drugs, or sub-optimal nutrition.

## Supporting Information

S1 Document Results of local health staff interviews regarding their perception of the newborn visual assessment. (PDF)

S1 File Vision assessment data. (SAV)

S1 Table Demographic & socio-economic characteristics of the parents of 179 newborns including pregnancy characteristics. (PDF)
