# Supplementary material for: Evaluation and Acceptability of a Simplified Test of Visual Function at Birth in a Limited-Resource Setting
Source: PLoS One. 2016 Jun 14;11(6):e0157087. doi: 10.1371/journal.pone.0157087 (PMC4907442; doi:10.1371/journal.pone.0157087)
Supplement: S1 Document — (PDF) [file pone.0157087.s001.pdf]

S1 Document: Results of local health staff interviews regarding their perception of the newborn visual assessment

The following open-ended questions were asked:

1. Is there a time/age when you prefer to do the test and why?
2. Can you tell me what, in the entire test, is the most difficult?
3. Can you tell me what, in the entire test, is the easiest?
4. What do you think about the test (like, don't like, usefulness)?

Transcript of the answers the local health staff provided

1. When asked if there was a preferred time/age when to do the test and why:

"*After doing the Dubowitz score, because the baby is not sleepy*" (Tester 3 and Tester 4)

"*At 24hrs the baby doesn't open the eyes and is not sucking well, at 48hrs the baby is more alert and has a better general condition*" (Tester 10)

"At 72 hrs the baby is more interested" (Tester 8)

Early (within 24 hours of age) *"because baby is not sleepy"* (Tester 5); "*because baby is easy to wake up"* (Tester 11); *"because baby is more awake"* (Tester 7); *"because the baby did not get vaccination yet so has no fever and doesn't cry"* (Tester 6)

"At 24-48 hrs of life, it is easier than at 72 hrs because baby is less sleepy" (Tester 1)

"Age doesn't matter if the child is awake" (Tester 2 and Tester 9)

2. When asked what is the most difficult part of the test:

"Difficult to deal with the child's change in behaviour" (Tester 6)

"Difficult to wake up the baby" (Testers 4, 5, 9)

"Difficult to keep the child awake" (Testers 2, 8)

"Difficult if the child is crying" (Tester 3)

"Difficult if the child is hungry" (Tester 7)

"Difficult if the child is crying all the time, is hungry or is sleepy" (Tester 10)

"Difficult to assess the target and the stripes if the baby feels sleepy" (Tester 11)

"Difficult to do the test with a lot of noise or hot weather" (Tester 1)

3. When asked what is the easiest:

“Test itself is easy” (Testers 1, 2, 6, 7, 8, 9, 10)

“Looking at the eye movements” (Testers 3, 4, 11)

“Testing the attention to a colour stimulus” (Tester 5)

4. When asked their thoughts about the test in terms of what they like about it, don’t like, find useful (or not):

“When the baby wakes up and we can complete the test, we can explain to the mother that the baby can see everything that we have shown to her and this is good because some mothers say the baby sees the mother face only at 4 months of age” (Tester 11)

“I can see if the baby can see or not, so it is interesting and it makes parents happy” (Tester 3)

“I like to do the target cards” (Tester 9)

“I know the vision; I can see it and this is interesting” (Tester 2)

“Useful test for abnormal eye babies because we can follow-up the child and see if the baby improves” (Tester 1)

“Useful test for the baby and easy test to learn and also to teach” (Tester 4)

“The test time is short and the test is easy to remember” (Tester 5)

“We can see if the baby has eye problems early on, like nystagmus or strabismus” (Tester 7)

“We can know the vision of the eye early, if it is normal or abnormal” (Tester 10)

“When the weather is hot and there is noise around, the test is difficult and same when the baby is sleepy” (Tester 1)

“I don’t like to disturb the babies when they are feeling sleepy” (Tester 11)

“Some parents are not easy to negotiate with” (Tester 2)

“The time to wait to have a good behaviour of the baby is not a nice part” (Tester 6)
