# Supplementary material for: Evaluation and Acceptability of a Simplified Test of Visual Function at Birth in a Limited-Resource Setting
Source: PLoS One. 2016 Jun 14;11(6):e0157087. doi: 10.1371/journal.pone.0157087 (PMC4907442; doi:10.1371/journal.pone.0157087)
Supplement: S1 Table — (PDF) [file pone.0157087.s003.pdf]

S1 Table. Demographic & socio-economic characteristics of the parents of 179 newborns including pregnancy characteristics

Characteristics	Mother	Father
Age (in years)	25.4 ± 6.1 [16-43]	29.7 ± 7.5 [15-51]
< 18 years old (adolescents)	4 (2.2%)	2 (1.1%)
> 40 years old	1 (0.6%)	16 (8.9%)
Ethnicity Karen*	146 (81.6%)	146/178 (82.0%)
Duration of residence at current address along the Thailand-Myanmar border (in years)	10 [<1-37]	10 [<1-45]
Did attend school*	123 (68.7%)	134/178 (75.3%)
School level reached		
Primary level	63/123 (51.2%)	61/134 (44.8%)
Secondary level	60/123 (48.8%)	65/134 (48.5%)
Higher education level	0 (0%)	1/134 (0.7%)
Religious or non-government school system	0 (0%)	8/134 (6.0%)
Can read	125 (69.8%)	143 (79.9%)
Smoking*	33 (18.4%)	121/178 (68.0%)
Drinking alcohol ≥ 5 days a week*	0 (0%)	21/178 (11.8%)
**Pregnancy characteristics**		**Not applicable**
Trimester at enrolment to antenatal care		
1st trimester	75 (41.9%)	
2nd trimester	76 (42.5%)	
3rd trimester	28 (15.6%)	
Gravidity	2 [1-9]	
Parity	1 [0-7]	
Primipara	46 (25.7%)	

Grand multipara (> 4 live-births prior to this child)	9 (5.0%)
Height (cm)	151.1 ± 5.2 [137-175]
< 145cm	14 (7.8%)
Weight on admission (kg)	50.3 ± 7.4 [34-85]
Enrolled at 1st trimester	48.7 ± 7.0 [35-69]
Enrolled at 2nd trimester	50.5 ± 7.5 [34-85]
Enrolled at 3rd trimester	54.3 ± 6.8 [46-71]
BMI (kg/m^2) for women enrolled in 1st trimester only	21.2 ± 2.8 [16.4-30.3]
< 18.5 kg/m^2	11 (14.7%)
18.5 to 22.9 kg/m^2	51 (68.0%)
≥ 23.0 kg/m^2	13 (17.3%)
Weight at delivery (if taken within 10 days of birth) (kg)*	57.4 ± 7.2 [41-92]
Anemia (Hematocrit < 30% at least once in pregnancy)	30 (16.8%)

Numbers are mean ±SD, [range] or median [range] or number (%)

*1 father information missing; 16 weight measurements at delivery missing
